# Assessing affective states in domestic dogs through judgment bias tasks: a systematic review of methods, influencing factors, and welfare applications

**DOI:** 10.3389/fvets.2026.1926433

**Published:** 2026-10-23

**Authors:** Valentina Gazzano, Luca Ciurli, Maria Claudia Curadi, Paolo Baragli, Angelo Gazzano, Asahi Ogi

**Affiliations:** 1 Department of Veterinary Sciences, University of Pisa, Pisa, Italy; 2 Department of Clinical Sciences and Translational Medicine, University of Rome Tor Vergata, Rome, Italy

**Keywords:** affective states, animal welfare, canine, cognitive bias, dog, emotional assessment, judgment bias, review

## Abstract

**Background:**

Judgment bias tasks (JBTs) have emerged as a promising approach for assessing affective states in animals, including domestic dogs, by measuring optimistic or pessimistic responses to ambiguous stimuli as proxies for underlying emotion. Despite growing use, the canine evidence base had not been systematically evaluated.

**Methods:**

We conducted a systematic review following PRISMA 2020 guidelines, searching Scopus and PubMed, with a supplementary search of Web of Science Core Collection. Thirty-two studies (15 experimental, 13 observational/quasi-experimental, 4 methodological) met the inclusion criteria. Experimental studies were appraised with the SYRCLE risk-of-bias tool. Findings were synthesized narratively, as methodological heterogeneity precluded meta-analysis.

**Results:**

Five paradigm types were identified, but 28 of the 32 studies (88%) used the same spatial discrimination task; the four remaining paradigms were represented by one study each. JBTs showed sensitivity to some experimental manipulations, including oxytocin (*d* = 2.16) and environmental enrichment (nosework, *p* < 0.01), while observational and quasi-experimental studies also reported associations between training method and JBT performance (e.g., aversive versus reward-based training: *Z* = −2.38, *p* = 0.017). However, validity and reliability concerns were pervasive: ceiling effects at ambiguous locations, confounding by spatial-learning ability, poor test–retest reliability, and absent protocol standardization. Convergence with independent welfare indicators was only partial.

**Conclusion:**

JBTs are responsive to some affective manipulations and are associated with relevant welfare and experiential factors, but substantial validity and reliability limitations remain. Standardized protocols and multi-method validation are needed before JBTs can serve as field measures of canine welfare.

## 1 Introduction

### 1.1 Animal welfare and affective states

The assessment of animal welfare has long been predicated upon the evaluation of physiological stress indicators, behavioral expressions, and health outcomes (1, 2). However, contemporary welfare science increasingly recognizes that subjective emotional experiences constitute a fundamental dimension of wellbeing (3–5). The assessment of positive emotions, in particular, has been identified as essential for a comprehensive evaluation of animal welfare that goes beyond the mere absence of suffering (4).

In domestic dogs (*Canis familiaris*), welfare encompasses not only the absence of negative physical states such as pain or disease, but also the presence of positive emotional experiences and the reduction of aversive psychological states such as fear, anxiety, and frustration (4). This paradigm shift reflects mounting evidence that animals possess conscious experiences, and that the quality of these experiences meaningfully affects their overall welfare status (6).

Accurately measuring affective states in non-human animals presents a methodological challenge, given their inability to provide direct verbal reports of internal emotional experiences. Consequently, researchers have developed indirect measures—behavioral, physiological, and cognitive—that serve as proxies for affective state assessment (7). Conventional behavioral indicators such as play, social interaction, and exploratory behavior are readily observable and have contributed substantially to welfare science (8). Physiological markers including cortisol concentration (measured via fecal, salivary, or blood assays), heart rate variability, and immunological measures provide quantifiable indices of stress-related activation (9). Nevertheless, these traditional approaches possess inherent limitations: they may reflect acute physiological responses rather than sustained affective states, they are often context-dependent, and they do not directly measure the subjective experience of emotion itself. Cognitive approaches, by contrast, offer the potential to assess affective states through measurable biases in decision-making and perception—domains that theory suggests are fundamentally influenced by emotional valence.

### 1.2 The cognitive bias framework and the judgment bias paradigm

The cognitive bias framework draws upon established principles from human cognitive psychology, where affective states systematically influence information processing, attention, memory, and judgment (10, 11). Cognitive bias is an umbrella concept encompassing systematic affect-related changes across several domains of information processing, including attention, memory, perception, and judgment (10, 11). Within this broader framework, judgment bias specifically refers to the tendency to interpret ambiguous information according to an individual’s underlying affective state, with more positive states generally associated with expectations of positive outcomes and more negative states with expectations of negative outcomes (12). Judgment bias tasks (JBTs) operationalize this specific form of cognitive bias by assessing responses to ambiguous stimuli after subjects have learned associations between reference stimuli and relatively positive or negative outcomes (12). Thus, judgment bias can be considered a specific manifestation of the broader cognitive bias framework rather than a separate theoretical framework. While JBTs share with other cognitive-bias approaches the potential to provide indirect insight into affective states, they also involve specific methodological challenges related to discrimination learning, responses to ambiguous probes, repeated testing, and task-specific sensory or cognitive confounds. The application of this approach to non-human animals was first experimentally demonstrated by Harding et al. (12), using rats (*Rattus norvegicus*). Rats trained to press a lever in response to one tone for food reward and another tone to avoid a negative event (30 s of 70 dB white noise) showed pessimistic responses to ambiguous intermediate tones when housed in unpredictable conditions. Following this seminal work, the judgment bias paradigm has been applied across a range of species, providing converging evidence that affective states modulate cognitive processing in animals. In sheep, Doyle et al. (13) demonstrated that release from restraint generated a positive judgment bias, while administration of a serotonin inhibitor induced pessimistic-like responses (14). In pigs, environmental enrichment has been shown to induce optimistic cognitive biases (15), while in horses, increased pessimistic responses have been linked to sub-optimal environmental conditions, such as constraints related to social interaction, space, and feeding (16).

The paradigm has also been extended to aquatic species: recent studies on zebrafish (*Danio rerio*) have shown that the cognitive bias test can effectively detect positive and negative affective states in this species (17), and that physical environmental enrichment significantly reduces stress responses upon exposure to novel conditions (18). This growing body of cross-species evidence supports the theoretical validity of cognitive bias as an indicator of animal emotion and welfare (19). These cross-species findings are reported here as they were presented by their original authors. They were identified through a non-systematic search and were not appraised against the criteria applied to the canine corpus in this review; with the exception of dedicated validation studies, they are subject to methodological limitations comparable to those documented below for dogs.

### 1.3 Judgment bias tasks in domestic dogs: development and current landscape

The application of judgment bias paradigms to domestic dogs commenced with the influential work of Mendl et al. (20). This study demonstrated the feasibility of judgment bias testing in dogs and provided preliminary evidence for its validity as an emotional assessment tool. Since then, the literature on canine judgment bias tasks (JBTs) has expanded substantially, with researchers adopting diverse methodological approaches including visual discrimination paradigms [see (21)], auditory discriminations [see (22)], Go/No-Go tasks on touchscreen devices [see (23)], and modified spatial paradigms with increased numbers of ambiguous locations [see (24)]. This diversity of approaches has enriched the field by allowing investigation of affective states across multiple sensory modalities and decision contexts, yet it has simultaneously created challenges for comparing findings across studies and synthesizing evidence.

Dogs are an important study population for welfare science because they are kept under a wide range of human-imposed conditions that carry recognized welfare risks: confinement in shelters and kennels, aversive training practices, separation from owners, restricted opportunity for species-typical behavior, and selective breeding for conformational traits associated with chronic disease. Their close and prolonged contact with humans, and their accessibility for systematic research, make these welfare challenges unusually tractable to study rather than being reasons in themselves to prioritize the species. The heterogeneous housing and management conditions under which dogs live, ranging from family homes to shelters to working and breeding establishments, create natural variation in welfare conditions that allows investigation of how affective states correlate with environmental and experiential factors.

Furthermore, the growing public interest in dog welfare means that validated measurement tools could support improvements in animal management and policy frameworks. Consequently, the development of efficient, reliable, and valid measures of affective state in dogs is a priority for veterinary and applied animal behavior science.

### 1.4 Research questions and rationale for systematic review

Despite the theoretical appeal of judgment bias tasks for assessing affective states in dogs, and the accumulation of empirical studies since 2010, the evidence base has not been systematically synthesized or critically evaluated. To our knowledge, the present work constitutes the first systematic review focused specifically on judgment bias tasks in domestic dogs; while a broader narrative literature has examined cognitive bias as an indicator of animal emotion across species (19), no prior synthesis has critically and systematically evaluated the canine evidence base. This gap is of practical as well as scientific importance: validated JBT tools could inform welfare-related policy and practice across the heterogeneous contexts in which dogs live, including shelter housing standards, training-method regulations, breeding practices, and the assessment of working and medical detection dogs. Moreover, more than half of the studies included in the present review (22 of 32) were published between 2018 and 2025, indicating that the field has entered a phase of rapid expansion in which the volume of empirical work is outpacing critical synthesis. A species-specific synthesis is also warranted because canine JBT paradigms face distinctive challenges that broader cross-species reviews do not address: the cognitive variability introduced by domestication and selective breeding, the diversity of housing and management contexts (from family homes to shelters and working environments), and the direct relevance of the findings to applied welfare science.

Key unresolved questions persist regarding the validity of JBTs as measures of affect, the reliability of task performance, the influence of methodological factors (paradigm design, stimulus characteristics, outcome metrics) on results, and the true magnitude of effects of experimental and naturalistic affective state manipulations. Moreover, the extent to which JBT performance converges with established welfare indicators, and the degree to which it predicts or correlates with behaviorally and physiologically defined affective states, remains incompletely characterized.

This systematic review addresses two overarching research questions:

*RQ1*: Does the JBT provide valid and reliable evidence of affective state in dogs, and what methodological factors influence task performance?

*RQ2*: Do deliberate experimental manipulations of experience and affective state produce measurable shifts in JBT performance?

These questions were selected to directly address the foundational validity and sensitivity of the approach.

By systematically reviewing the available evidence, we aim to synthesize findings regarding the responsiveness of JBTs to affective manipulations, critically evaluate methodological quality and risk of bias, identify sources of heterogeneity in task design and outcomes, evaluate the strength of evidence for convergent validity with independent welfare measures, and provide evidence-based recommendations for future methodological development and application.

The ultimate goal is to characterize the current state of knowledge regarding judgment bias testing in dogs and to delineate a path forward for improving the scientific rigor and practical utility of the approach.

## 2 Methods

### 2.1 Study design

This work constitutes a systematic review of the literature on judgment bias tasks in domestic dogs. The methodology was designed and reported in accordance with the Preferred Reporting Items for Systematic Reviews and Meta-Analyses (PRISMA) 2020 guidelines (25).

Although a systematic search strategy was undertaken and studies were subject to pre-specified eligibility criteria and critical appraisal, we adopted a narrative synthesis approach rather than meta-analytical pooling, as discussed below.

### 2.2 Eligibility criteria

Eligible studies were required to: (1) include domestic dogs (*Canis familiaris*) as study subjects; (2) employ a judgment bias task or closely related cognitive bias paradigm; (3) be published in English in a peer-reviewed journal; and (4) provide sufficient methodological detail to confirm the use of an eligible judgment bias paradigm and sufficient outcome data to extract findings relevant to the review questions.

Studies were included if they provided information relevant to either of the two research questions of the present review (RQ1 and RQ2). These are the questions of this review; they were not necessarily the questions posed by the original authors, and no study was required to have addressed them as such. No restrictions were imposed based upon publication date, dog breed, age, housing condition, or sample size, to ensure comprehensive coverage of the field. Studies addressing cognitive biases in domains other than judgment/decision-making (e.g., attentional bias, memory bias) were included if the study also employed a judgment bias measure; purely attentional or memory studies were excluded unless they directly measured judgment bias outcomes.

### 2.3 Search strategy and study selection

A comprehensive literature search was conducted in March 2026 using two major databases: Scopus and PubMed. The search strategy employed Boolean operators and subject-specific terminology to maximize sensitivity whilst maintaining acceptable specificity. The core search string was: TITLE-ABS-KEY ((judgment OR cognitive) AND bias AND (dog OR canine)) AND (LIMIT-TO (LANGUAGE, “English”)). This was adapted slightly for PubMed syntax: (“judgment bias”[tiab] OR “judgement bias”[tiab] OR “Cognitive Biases”[MeSH] OR “cognitive bias”[tiab] OR “cognitive biases”[tiab]) AND (dog[tiab] OR canine[tiab] OR “Dogs”[MeSH]).

A supplementary search of Web of Science Core Collection was subsequently conducted using an equivalent search strategy to assess the completeness of database coverage. This search did not identify additional eligible studies published within the original search period beyond those already retrieved through Scopus and PubMed.

The search was confined to peer-reviewed literature. Initial searches yielded 150 records from Scopus and 39 records from PubMed, for a total of 189 records prior to deduplication. After removal of duplicates 156 unique records remained. Full-text screening of articles was conducted independently by two reviewers. They achieved an almost perfect agreement, with 151 observed agreements and a kappa score of 0.905. A third reviewer evaluated the 5 articles where disagreement occurred. Ultimately, 32 studies were selected for inclusion in the final systematic review (Figure 1).

**Figure 1:**
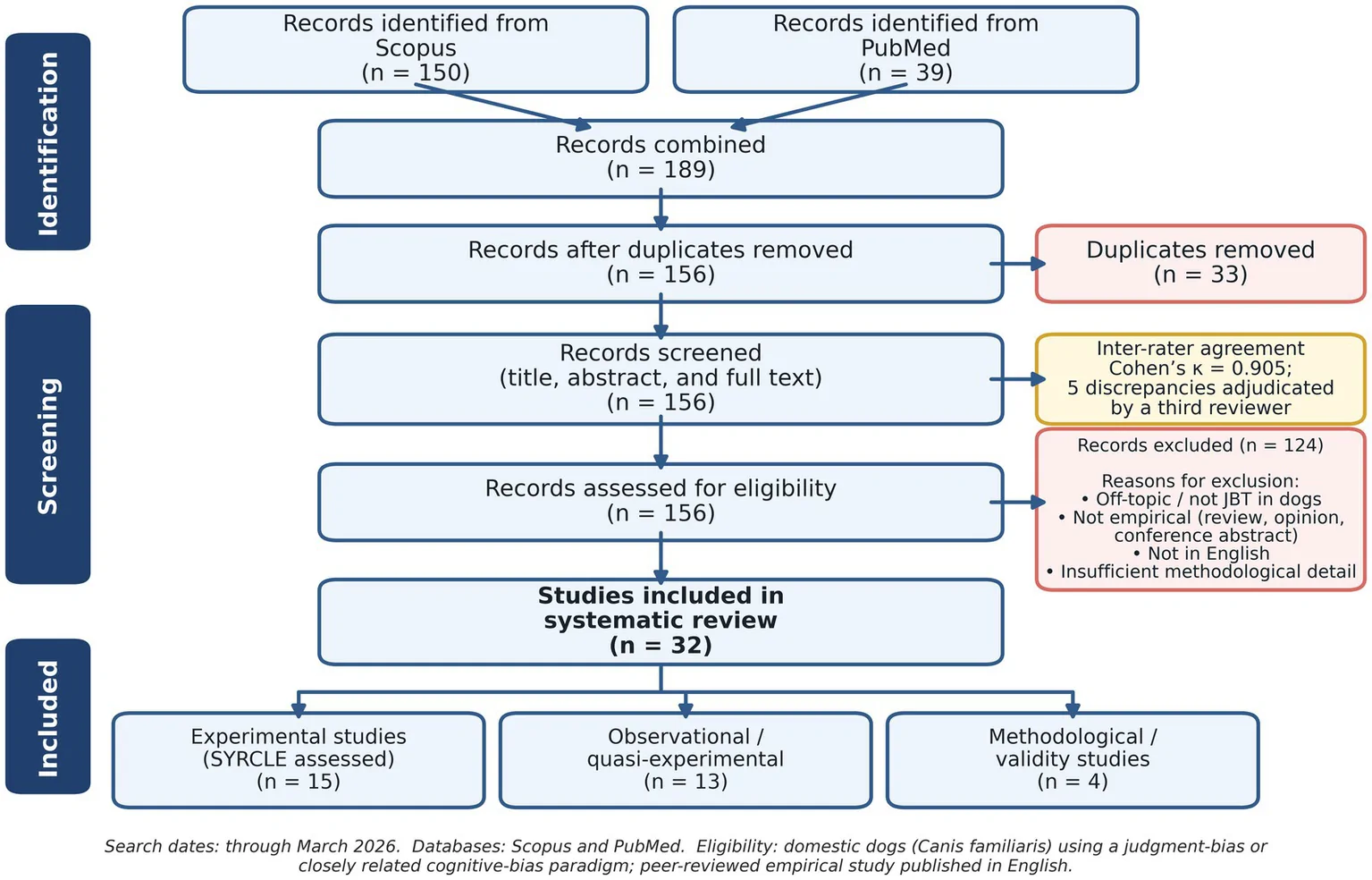
PRISMA 2020 flow diagram.

### 2.4 Data synthesis and justification for absence of meta-analysis

Findings were synthesized narratively, organized by thematic domain (paradigm typology, validity and reliability, experimental manipulations, observational associations, and convergent validity). A formal meta-analysis was considered but deemed inappropriate following a systematic assessment of the included studies, due to high methodological and statistical heterogeneity across the corpus.

Three principal sources of heterogeneity precluded meaningful pooling. First, heterogeneity in the way the judgment bias task was implemented and scored. This is not primarily heterogeneity between paradigms: 28 of the 32 studies used a spatial discrimination task, and the visual, auditory, Go/No-Go and attention-bias paradigms were represented by one study each. It is heterogeneity within the dominant spatial paradigm. As set out in Tables 1–4, the number of trained and ambiguous locations varied from three to five; the outcome variable was variously the raw approach latency, a latency or running speed rescaled to each dog’s own positive and negative locations, a log-transformed latency, or a within-dog pre-post difference score; and the maximum trial duration at which non-approach was censored ranged from 10 to 30 s. A rescaled score and a raw latency in seconds are not on a common metric, and differing censoring thresholds truncate the distributions differently, so these outcomes cannot be combined without re-analysis of individual-level data, which were not available for most studies.

**Table 1:** Paradigm-defining studies referenced in section 3.2.1.

References	*N*	Study design	Population	Paradigm (trained + ambiguous locations)	Latency measure and adjustment for baseline running speed	Statistical approach, effect sizes and descriptive statistics	Key finding
Mendl et al. (20)	24	Case–control	Shelter dogs	Spatial; 2 trained + 3 ambiguous	Mean latency per location, adjusted to each dog’s mean training-trial P/N baseline latencies; raw latencies also analyzed	Friedman test and Spearman correlations with SRB score; rs reported; latency descriptives graphical (means ± SEM)	Foundational spatial paradigm; SRB dogs showed pessimistic bias
Burman et al. (21)	12	Within-subjects	Lab Beagles	Visual greyscale; 2 trained cards + 1 intermediate card	Latency to goal box (30 s ceiling), rescaled to each dog’s rewarded/unrewarded reference latencies, log-transformed	Repeated-measures GLMs with *post hoc* paired *t*-tests; partial eta-squared reported; latency descriptives graphical (means ± SEM)	Visual grayscale paradigm developed; slower approach post-consumption
Starling et al. (22)	20	Experimental (auditory)	Pet dogs	Auditory tones; 2 trained tones + 9 ambiguous probe tones	Raw latency to touch the target, censored at 10 s; no speed adjustment (the response is a target touch, not an approach); derived tipping-point and latency-variability indices	Cox proportional hazards regression; no effect sizes reported; latency descriptives largely graphical	Auditory tone paradigm; tipping-point and survival-analytic outcomes introduced
Bunford et al. (23)	29	Cross-sectional	Family dogs	Go/No-Go touchscreen; 2 trained stimuli + 2 ambiguous stimuli	Primary outcome is the proportion of “go” responses, not latency; go-response latency recorded raw and untransformed; speed adjustment not applicable	Multivariate linear regression with backward elimination; Cox regression for latency; partial eta-squared reported (0.19; 0.14); descriptives tabulated	Go/No-Go touchscreen paradigm with colored shape stimuli
Burani et al. (24)	50	Methodological	Pet dogs	Spatial, five-corridor variant; 2 trained + 3 ambiguous	Raw, untransformed trial-level latencies (15 s ceiling); primary LMM not speed-adjusted; Mendl-adjusted score (0–100) as secondary measure	LMMs (maximum likelihood; corridor nested in dog) with BIC model selection; non-parametric ancillary tests; no standardized effect sizes; means ± SD reported	Five-corridor spatial variant; 98% acquisition, improved discrimination
Hobbs et al. (32)	68	Case–control	IE dogs + controls	Spatial; 2 trained + 3 ambiguous, plus a separate auditory attention-bias task	Raw latency (s), analyzed unadjusted; a score rescaled to each dog’s own positive and negative latencies also computed. Non-adjustment justified by matching groups on breed, age and sex and standardizing arena size	Linear mixed models with dog identity as random effect; Friedman with Dunn-Bonferroni; no effect sizes; latency descriptives mainly graphical	Combined spatial JBT with attention-bias task as complementary paradigms

Column definitions: N is the number of dogs as reported by the study authors. Paradigm gives the task type with the number of trained and ambiguous locations. Latency measure states the outcome variable analyzed and whether it was adjusted for the individual dog’s baseline running speed. Statistical approach states the model or test used and whether standardized effect sizes and numerical descriptive statistics were reported. ANOVA, analysis of variance; BIC, Bayesian information criterion; CI, confidence interval; GLM, general linear model; IE, idiopathic epilepsy; JBT, judgment bias task; Lab, laboratory-housed; LMM, linear mixed-effects model; P/N, positive/negative reference locations; RCT, randomized controlled trial; SD, standard deviation; SE, standard error; SEM, standard error of the mean; SRB, separation-related behavior.

**Table 2:** Studies of validity and reliability synthesized in section 3.2.2.

References	N	Study design	Population	Paradigm (trained + ambiguous locations)	Latency measure and adjustment for baseline running speed	Statistical approach, effect sizes and descriptive statistics	Key finding
Gruen et al. (26)	16	Methodological (JBT + DNMP)	Lab Labradors	Spatial; 2 trained + 3 ambiguous [as replicated by Wilson et al. (28)]	Per-position mean latencies (30 s ceiling); unadjusted means plus Mendl-adjusted score rescaled to own P/N baseline latencies	Matched-pairs *t*-tests and Wilcoxon; Spearman correlations with DNMP acquisition (r^2^ reported); means ± SEM reported	Spatial-learning ability strongly predicts JBT latencies (*r*^2^ = 0.56, *p* = 0.001)
Burani et al. (27)	91	Methodological	Shelter + pet dogs	Spatial; 2 trained + 3 ambiguous	Raw, untransformed, trial-level latency (30 s ceiling) as the primary measure; a rescaled score (0–100, anchored on each dog’s own positive and negative latencies) analyzed in parallel. No separate baseline running-speed measure: dog identity as a random effect is used instead, and the authors argue against averaged rescaled scores	Linear mixed-effects models (maximum likelihood); Wilcoxon, Mann–Whitney and chi-squared tests; effect sizes for the chi-squared tests only (Cramer’s *V* = 0.07 and 0.22); means with SD reported	Raw vs. adjusted latencies yield divergent conclusions; weak adjacent-location discrimination
Burani et al. (24)	50	Methodological	Pet dogs	Spatial, five-corridor variant; 2 trained + 3 ambiguous	Raw, untransformed trial-level latencies (15 s ceiling); primary LMM not speed-adjusted; Mendl-adjusted score (0–100) as secondary measure	LMMs (maximum likelihood; corridor nested in dog) with BIC model selection; non-parametric ancillary tests; no standardized effect sizes; means ± SD reported	Five-corridor design improves acquisition (98%) and discrimination
Wilson et al. (28)	15	Repeated measures	Mixed (kennel + pet)	Spatial; 2 trained + 3 ambiguous	Latency log10-transformed; actual (unadjusted) latencies used, explicitly because dog identity was included in the model to absorb individual differences in relative speed	Linear mixed models for latency; generalized linear mixed model (logit) for approach/no-approach; intraclass correlation coefficients for reliability; no effect sizes; descriptives tabulated	Poor test–retest ICC (0.009–0.333); progressive pessimistic drift across sessions
Krahn et al. (33)	16	Quasi-experimental	Companion dogs	Spatial; 2 trained + 3 ambiguous	Raw latency to cross a 1 m circle around the bowl, rounded to the nearest second, 10 s ceiling; no adjustment applied and none discussed	Mixed model (SAS Proc Mixed, compound symmetry); no effect sizes; means with SD or SE reported	Pre-session discrimination training does not bias subsequent JBT (*p* = 0.86)

Column definitions: N is the number of dogs as reported by the study authors. Paradigm gives the task type with the number of trained and ambiguous locations. Latency measure states the outcome variable analyzed and whether it was adjusted for the individual dog’s baseline running speed. Statistical approach states the model or test used and whether standardized effect sizes and numerical descriptive statistics were reported. ANOVA, analysis of variance; BIC, Bayesian information criterion; CI, confidence interval; DNMP, delayed non-match to position task; ICC, intraclass correlation coefficient; JBT, judgment bias task; Lab, laboratory-housed; LMM, linear mixed-effects model; P/N, positive/negative reference locations; RCT, randomized controlled trial; SD, standard deviation; SE, standard error; SEM, standard error of the mean.

**Table 3:** Studies contributing evidence on manipulations and interventions synthesized in section 3.2.3 (grouped by manipulation type).

References	N	Study design	Population	Paradigm (trained + ambiguous locations)	Manipulation type	Latency measure and adjustment for baseline running speed	Statistical approach, effect sizes and descriptive statistics	Direction and key statistic
Kis et al. (30)	64	Between-subjects 2 × 2 RCT	Pet dogs	Spatial; 2 trained + 1 ambiguous	Pharmacological	Positive expectancy score: the ambivalent-location latency rescaled to each dog’s own positive and negative latencies. Adjustment explicitly justified by large individual variation in running speed	Generalized estimating equations and general linear model with planned *t*-tests; Cohen’s *d* reported for all contrasts; means with SD and range tabulated	Optimistic shift; *d* = 2.16 (comm.), 0.97 (non-comm.); *p* < 0.001
Karagiannis et al. (43)	12	Pre-post quasi-experimental	SRP dogs + controls	Spatial; 2 trained + 3 ambiguous	Pharmacological	Running speed (m/s) converted to an adjusted score (0–100) anchored on the mean speed to the positive and negative locations at the end of training. Adjustment justified by differences in body size and food motivation	Non-parametric (Mann–Whitney, Wilcoxon signed-rank, Kendall’s tau); effect size r reported for each test; medians with range tabulated	Bias normalized after fluoxetine + behavior modification (*p* < 0.05)
Vieira de Castro et al. (34)	92	Quasi-experimental	Pet dogs	Spatial; 2 trained + 3 ambiguous	Training method	Raw latency (s), 20 s ceiling; no adjustment, individual differences handled only by including dog as a random effect	Generalized linear mixed model (SAS GLIMMIX); one Cohen’s *d* reported (aversive vs. reward-based), none for the other contrasts; latency descriptives graphical only	Aversive training → pessimistic bias + cortisol elevation (*p* < 0.05)
Casey et al. (35)	100	Matched-pair case–control	Pet dogs	Spatial; 2 trained + 3 ambiguous	Training method	Raw latency (s), 30 s ceiling; explicitly reported as unadjusted, with no rationale given	Non-parametric throughout (Wilcoxon matched-pairs on pair-matched dogs; Mann–Whitney); no effect sizes; latency descriptives graphical only	Aversive training → slower middle (*Z* = −2.38, *p* = 0.017)
Cimarelli et al. (44)	30	Between-subjects RCT	Pet dogs	Spatial; 2 trained + 3 ambiguous	Training schedule	Latency score = (probe latency − mean positive latency) × 100/(mean negative latency − mean positive latency), anchored on standard trials within the test session. Adjustment explicitly justified by individual differences in running speed	Linear mixed models (lme4); no standardized effect sizes (estimates with SE and t); medians with SD reported in text	Partial reinforcement → pessimistic bias (*p* < 0.05)
Duranton and Horowitz (36)	20	RCT	Pet dogs	Spatial; 2 trained + 1 ambiguous	Enrichment (nosework)	Raw latency (s), analyzed as a within-dog pre/post difference score, which the authors chose in order to control for individual speed differences rather than rescaling to the positive and negative locations	Fisher-Pitman permutation tests; Cohen’s *d* with 95% CI reported; means with SE reported	Optimistic shift; *d* = 0.64; *p* < 0.01
Uccheddu et al. (45)	110	Between-subjects RCT	Shelter dogs	Spatial; 2 trained + 1 ambiguous (middle; shortened Mendl protocol)	Enrichment (olfactory)	Raw latency to bowl (30 s ceiling); two-trial means per location; not rescaled to own P/N latencies	Wilcoxon signed-rank (before vs. after exposure) and paired *t*-tests; no effect sizes; means and SD reported	Oil blend → reduced ambiguous latency (*p* = 0.039); lavender → cortisol only
Willen et al. (37)	124	Quasi-experimental	Shelter dogs	Spatial; 2 trained + 3 ambiguous (NP, M, NN)	Enrichment (human)	Latency to bowl (30 s scored if unreached), expressed as percent of each dog’s own P–N latency difference (running-speed control)	Factorial and repeated-measures ANOVAs (Treatment × Fearfulness × Sex); no standardized effect sizes; descriptives graphical (means ± SE)	Fearful dogs: optimistic shift (*p* < 0.01); non-fearful: opposite (*p* < 0.05)
Chan et al. (38)	47	RCT	Companion dogs	Spatial; 2 trained + 1 ambiguous	Enrichment (toys)	Raw latency (s) as the primary measure, analyzed as a pre/post change; a Mendl-type rescaled latency reported in parallel, with the formula given only in supplementary material	Non-parametric (Wilcoxon with Pratt correction; Kruskal-Wallis); Glass rank-biserial effect sizes with CIs; means with SD reported despite non-parametric testing	No effect (*p* = 0.905)
Parr-Cortes et al. (31)	18	Within-subjects, double-blinded	Pet dogs	Spatial; 2 trained + 3 ambiguous	Olfactory (human odor)	Raw latency (s), censored at 30 s and analyzed as time-to-event; no rescaling, with dog identity as a random effect stated to control for individual running speed	Cox proportional hazards mixed model; hazard ratios reported as effect sizes; descriptives partly graphical	Stress odor → pessimistic shift at near-negative location
Müller et al. (39)	24	Within-subjects	Pet dogs	Spatial; 2 trained + 3 ambiguous (NP, M, NN)	Social (owner presence vs. absence)	Latency to bowl (30 s max) rescaled to own P/N latencies (0–100 score), log-transformed; adjusted for running speed	LMMs (treatment, location, test order; dog as random effect); no standardized effect sizes; latency-score descriptives graphical	Null; *F*(1,537) = 0.04, *p* = 0.84 (owner presence); longer latencies on day 2: *F*(1, 537) = 25.5, *p* < 0.001
Walker et al. (40)	12	Within-subjects	Shelter dogs (paired)	Spatial; 2 trained + 3 ambiguous (NP, M, NN)	Social (separation)	Latency to bowl (30 s max); speed-adjusted scores computed but unadjusted latencies analyzed (adjustment did not alter significance)	Friedman with post hoc Wilcoxon signed-rank (sequential Bonferroni); Cohen’s r reported; JBT latency descriptives graphical	No JBT effect (*p* = 0.33) despite physiological/behavioral change
Kinnaird and Wells (41)	45	Between-subjects RCT	Pet dogs	Spatial; 2 trained + 3 ambiguous (NP, M, NN)	Auditory	Latency to bowl (30 s max); adjusted score = percent of own P–N latency difference; unadjusted latencies also tabled	Non-parametric (Kruskal-Wallis, Friedman, Mann–Whitney, Wilcoxon); r reported for Mann–Whitney only; means ± SEM and 95% CI reported	No effect on bias (*p* > 0.05); location gradient confirmed
Burman et al. (21)	12	Within-subjects	Lab Beagles	Visual greyscale; 2 trained cards + 1 intermediate card	Reward (food)	Latency to goal box (30 s ceiling), rescaled to each dog’s rewarded/unrewarded reference latencies, log-transformed	Repeated-measures GLMs with post hoc paired *t*-tests; partial eta-squared reported; latency descriptives graphical (means ± SEM)	Counterintuitive: post-reward pessimism in visual paradigm
Starling et al. (22)	20	Experimental (auditory)	Pet dogs	Auditory tones; 2 trained tones + 9 ambiguous probe tones	Methodological/test	Raw latency to touch the target, censored at 10 s; no speed adjustment (the response is a target touch, not an approach); derived tipping-point and latency-variability indices	Cox proportional hazards regression; no effect sizes reported; latency descriptives largely graphical	Declining response rate across sessions (habituation/learning)

Column definitions: N indicates the total number of dogs participating in the study, as reported by the study authors; where outcome-specific exclusions occurred, the analyzed sample may be smaller. Paradigm gives the task type with the number of trained and ambiguous locations. Latency measure states the outcome variable analyzed and whether it was adjusted for the individual dog’s baseline running speed. Statistical approach states the model or test used and whether standardized effect sizes and numerical descriptive statistics were reported. ANOVA, analysis of variance; CI, confidence interval; comm., communicative context; GLM, general linear model; JBT, judgment bias task; Lab, laboratory-housed; LMM, linear mixed-effects model; M, middle (ambiguous) location; N, negative (unrewarded) location; NN, near-negative location; non-comm., non-communicative context; NP, near-positive location; P, positive (rewarded) location; P/N, positive/negative reference locations; RCT, randomized controlled trial; SD, standard deviation; SE, standard error; SEM, standard error of the mean; SRP, separation-related problems.

**Table 4:** Observational and quasi-experimental studies synthesized in section 3.2.4.

References	*N*	Study design	Population	Paradigm (trained + ambiguous locations)	Comparison (groups compared or predictor analyzed)	Latency measure and adjustment for baseline running speed	Statistical approach, effect sizes and descriptive statistics	Direction and key statistic
Mendl et al. (20)	24	Case–control	Shelter dogs	Spatial; 2 trained + 3 ambiguous	12 shelter dogs showing separation-related behavior vs. 12 shelter dogs not showing it	Mean latency per location, adjusted to each dog’s mean training-trial P/N baseline latencies; raw latencies also analyzed	Friedman test and Spearman correlations with SRB score; rs reported; latency descriptives graphical (means ± SEM)	SRB dogs more pessimistic; SRB frequency correlated with bias
Cockburn et al. (46)	21	Case–control	Cavalier King Charles Spaniels	Spatial; 2 trained + 3 ambiguous	8 Cavalier King Charles Spaniels with MRI-confirmed syringomyelia vs. 13 unaffected dogs of the same breed; analgesic use and owner-reported scratching score entered as additional terms	Latency to the bowl (30 s ceiling) rescaled as [(ambiguous − mean positive)/(mean negative − mean positive)] × 100, stated to control for individual running speed and arena size	Mixed-model ANOVA with scratching score as covariate; no effect sizes; latency descriptives graphical only	Syringomyelia → pessimistic bias (*p* < 0.05); resistant to analgesia
Hobbs et al. (32)	68	Case–control	IE dogs + matched controls	Spatial; 2 trained + 3 ambiguous, plus a separate auditory attention-bias task	Dogs with idiopathic epilepsy vs. controls matched on breed (Border Collies and Labrador Retrievers), age and sex	Raw latency (s), analyzed unadjusted; a score rescaled to each dog’s own positive and negative latencies also computed. Non-adjustment justified by matching groups on breed, age and sex and standardizing arena size	Linear mixed models with dog identity as random effect; Friedman with Dunn-Bonferroni; no effect sizes; latency descriptives mainly graphical	No JBT group effect (*p* = 0.658); attention bias detected post-aversive stimulus
Harvey et al. (47)	20	Cross-sectional	RSPCA shelter dogs	Spatial; 2 trained + 1 ambiguous	No groups: continuous predictor is the proportion of time spent awake but motionless in the home kennel; dogs also compared by origin (stray, seized, relinquished) for learning speed	Single-trial latencies (30 s ceiling); ambiguous latency rescaled to each dog’s own P/N latencies (PES), then dichotomized at median	Friedman/Wilcoxon, Mann–Whitney, chi-square and multivariable logistic regression on PES groups; no standardized effect sizes; medians (IQR) and means ± SD reported	No association of waking inactivity with JBT; ceiling effects (PES 96.7%)
Barnard et al. (48)	31	Cross-sectional	Pet dogs	Spatial; 2 trained + 3 ambiguous	No groups: continuous personality predictors from the Dog Mentality Assessment (DMA) and the Canine Behavioral Assessment and Research Questionnaire (C-BARQ)	Latency to contact the bowl (30 s ceiling), log-transformed; the mean of each dog’s positive and negative latencies entered as a covariate rather than rescaling the outcome, to control for size- and motivation-related differences in speed	Linear mixed models fitted separately per ambiguous location, dog identity as random effect; no standardized effect sizes (estimates with 95% CI); latency descriptives graphical only	Sociability and excitability predict optimistic bias (*p* < 0.05)
Bunford et al. (23)	29	Cross-sectional	Family dogs	Go/No-Go touchscreen; 2 trained stimuli + 2 ambiguous stimuli	No groups: continuous owner-rated personality dimensions	Primary outcome is the proportion of “go” responses, not latency; go-response latency recorded raw and untransformed; speed adjustment not applicable	Multivariate linear regression with backward elimination; Cox regression for latency; partial eta-squared reported (0.19; 0.14); descriptives tabulated	Conscientiousness/extraversion predict more “go” (optimistic) responses
Bistre Dabbah et al. (49)	58	Cross-sectional	Medical detection dogs	Spatial; 2 trained + 3 ambiguous	No groups: associations with demographic variables, 11 behavioral test components and detection-task sensitivity and specificity; trainee vs. qualified dogs also compared	Raw latency (30 s ceiling) plus an adjusted latency score controlling for each dog’s mean latencies to the positive and negative locations during testing	Non-parametric tests (Friedman, Wilcoxon, Spearman) with stepwise multiple regression; adjusted R-squared reported (18–24%); means, SDs and ranges reported	Optimism → task ability (*p* = 0.002); pessimism → detection specificity (*p* = 0.021)
Piotti et al. (50)	107	Cross-sectional	Family dogs (young vs. aged)	Spatial or feature-based; 2 trained + 1 ambiguous	Young (2.5–6.5 years) vs. old (8–14.5 years) family dogs; discrimination by location vs. by physical stimulus features as a second factor	Cognitive bias score = [(ambiguous - positive) × 100]/(negative - positive), 15 s ceiling, stated to control for differences in running speed	Wilcoxon rank-sum tests with alpha corrected to 0.025; effect size r reported; medians with quartiles tabulated	No bias difference between age groups; older dogs slower learners
Pogány et al. (51)	91	Cross-sectional	Pet dogs (5 breeds)	Spatial; 2 trained + 1 ambiguous	Overweight or obese (body condition score 3) vs. normal weight (score 2), scored by a veterinarian; obesity-prone vs. non-obesity-prone breed group as a second factor	Raw latency (30 s ceiling), non-approaches censored; no rescaling, all three trial types modelled jointly instead	Mixed-effects Cox models; hazard ratios with 95% CI reported; latency descriptives presented as survival curves only	Overweight dogs more pessimistic (*p* = 0.026)
Wells et al. (52)	30	Cross-sectional	Pet dogs	Spatial; 2 trained + 3 ambiguous	Left-pawed (*n* = 7), right-pawed (*n* = 10) and ambilateral (*n* = 13) dogs, classified from 100 paw uses in a food-extraction test; handedness index also analyzed as a continuous variable	Positive and negative locations analyzed as raw latencies; the three ambiguous locations analyzed as Mendl-type rescaled scores, applied because dogs differed in size and therefore in running speed	Kruskal-Wallis and Friedman tests with Pearson and Spearman correlations; Kendall’s W and r reported; rescaled-score descriptives graphical only	Left-pawed dogs more pessimistic (*r* = 0.66, *p* < 0.001)
Titulaer et al. (53)	53	Cross-sectional	Kenneled dogs	Spatial; 2 trained + 3 ambiguous	Short-term (1 week to 3 months) vs. long-term (more than 6 months) resident kenneled dogs	Mean raw latency per location (30 s ceiling); not rescaled to individual baseline (dog fitted as random effect)	LMM (dog nested in center as random effect) with post hoc pairwise comparisons; no standardized effect sizes; means ± SD reported	No JBT difference between short- and long-term kenneled dogs
Owczarczak-Garstecka and Burman (54)	17	Cross-sectional	Shelter dogs	Spatial; 2 trained + 3 ambiguous	No groups: judgment bias latency used as one of four predictors of sleep and resting variables	Latency to the middle probe (30 s ceiling), rescaled following Mendl et al. to account for size-related differences in running speed; formula not reproduced in the paper	Friedman test with Bonferroni-corrected pairwise Wilcoxon tests; multiple linear regression (R-squared and standardized beta reported); mean latencies with SD tabulated	Higher daytime resting → optimistic bias and convergent welfare indicators

Column definitions: N is the number of dogs as reported by the study authors. Paradigm gives the task type with the number of trained and ambiguous locations. Latency measure states the outcome variable analyzed and whether it was adjusted for the individual dog’s baseline running speed. Statistical approach states the model or test used and whether standardized effect sizes and numerical descriptive statistics were reported. The Comparison column states what characterizes the groups being compared, or the predictor analyzed where no groups were formed. ANOVA, analysis of variance; C-BARQ, Canine Behavioral Assessment and Research Questionnaire; CI, confidence interval; DMA, Dog Mentality Assessment; IE, idiopathic epilepsy; IQR, interquartile range; JBT, judgment bias task; LMM, linear mixed-effects model; MRI, magnetic resonance imaging; P/N, positive/negative reference locations; PES, positive expectancy score; RCT, randomized controlled trial; SD, standard deviation; SE, standard error; SEM, standard error of the mean; SRB, separation-related behavior.

Second, the interventions and study designs were highly diverse in nature and mechanism, ranging from pharmacological treatments and training protocols to olfactory and auditory stimulation, making any aggregate effect estimate conceptually difficult to interpret.

Third, statistical reporting was inconsistent across studies: many relied exclusively on inferential statistics without descriptive data, used non-parametric or survival-analytic frameworks incompatible with standard effect size extraction, or presented outcomes only in graphical form. The statistical approach used by each study, and whether it reported effect sizes and numerical descriptive statistics, are set out study by study in Tables 1–4, together with the latency measure used. Sample sizes were consistently small, and the subset of studies from which effect sizes could plausibly be extracted would be insufficient to produce a stable pooled estimate.

These constraints are compounded by well-documented methodological limitations of the spatial JBT itself (26–28), including variability in acquisition criteria, scoring procedures, and statistical frameworks, as well as evidence that latency outcomes are sensitive to spatial learning ability and repeated testing exposure independently of affective state. Taken together, these factors render pooling not merely technically difficult but conceptually questionable. Findings are therefore presented as a narrative systematic review structured by thematic domain, with individual effect sizes noted descriptively where reported by study authors.

### 2.5 Risk of bias assessment

Risk of bias was assessed using the SYRCLE risk-of-bias tool described by Hooijmans et al. (29), which evaluates 10 methodological domains: sequence generation, baseline similarity, allocation concealment, random housing, blinding of caregivers/investigators, random outcome selection, blinding of assessors, incomplete data handling, selective outcome reporting, and other bias sources. Each domain was rated as low risk (Y), high risk (N), or unclear (NC). The SYRCLE assessment was used as a design-specific critical appraisal and not as an additional eligibility criterion. All studies meeting the review inclusion criteria remained part of the narrative synthesis regardless of whether SYRCLE was applicable. The tool was applied only to studies involving experimental manipulation, including mixed-design studies with an experimental component, because several SYRCLE domains concern allocation, randomization, and blinding. Purely observational, correlational, and methodological studies were therefore not assessed using SYRCLE but remained fully included in the systematic review.

## 3 Results

### 3.1 Characteristics of included studies

The 32 included studies were published between 2010 and 2025, spanning 15 years of research on judgment bias in domestic dogs. The field developed across four broad phases (foundational work 2010–2013; paradigm diversification 2014–2016; applied welfare studies 2017–2019; methodological scrutiny and consolidation 2020–2025). Studies were conducted across multiple countries including the United Kingdom, Italy, Hungary, Austria, Canada, the United States, Australia, New Zealand, Portugal, Northern Ireland, and Israel. Sample sizes ranged from 12 to 124, with populations encompassing shelter dogs, pet dogs, working dogs, laboratory-housed dogs, and medical detection dogs. Studies were categorized into 15 experimental, 13 observational or quasi-experimental, and 4 methodological studies. The full characteristics of all included studies are presented in Table 5.

**Table 5:** List of all 32 included studies (chronological order).

References	Year	Journal	Title	Study category	Primary objective
Mendl et al. (20)	2010	Current Biology	Dogs showing separation-related behavior exhibit a “pessimistic” cognitive bias	Observational/quasi-experimental	Examine the association between separation-related behavior and judgment bias
Burman et al. (21)	2011	Applied Animal Behavior Science	Using judgement bias to measure positive affective state in dogs	Experimental	Test whether receiving a food reward influences subsequent judgment bias
Müller et al. (39)	2012	Animal Cognition	Brief owner absence does not induce negative judgement bias in pet dogs	Experimental	Test whether brief owner absence affects judgment bias in pet dogs
Titulaer et al. (53)	2013	Applied Animal Behavior Science	Cross sectional study comparing behavioral, cognitive and physiological indicators of welfare between short and long term kenneled dogs	Observational/quasi-experimental	Compare behavioral, cognitive and physiological welfare indicators in short- and long-term kenneled dogs
Starling et al. (22)	2014	PLoS ONE	Canine sense and sensibility: Tipping points and response latency variability as an optimism index in a canine judgement bias assessment	Experimental	Evaluate an auditory JBT and response measures as indicators of optimism
Walker et al. (40)	2014	Applied Animal Behavior Science	The effect of conspecific removal on the behavior and physiology of pair-housed shelter dogs	Experimental	Test whether temporary conspecific separation affects JBT performance and other welfare indicators
Karagiannis et al. (43)	2015	BMC Veterinary Research	Dogs with separation-related problems show a “less pessimistic” cognitive bias during treatment with fluoxetine and a behavior modification plan	Experimental	Examine changes in judgment bias during treatment for separation-related problems
Kis et al. (30)	2015	Hormones and Behavior	Oxytocin induces positive expectations about ambivalent stimuli (cognitive bias) in dogs	Experimental	Test whether intranasal oxytocin influences responses to ambiguous stimuli
Owczarczak-Garstecka and Burman (54)	2016	PLoS ONE	Can sleep and resting behaviors be used as indicators of welfare in shelter dogs?	Observational/quasi-experimental	Examine associations between sleep/rest behavior, JBT performance and other welfare indicators
Wells et al. (52)	2017	Journal of Comparative Psychology	Cognitive bias and paw preference in the domestic dog	Observational/quasi-experimental	Examine the association between paw preference and judgment bias
Barnard et al. (48)	2018	Scientific Reports	Personality traits affecting judgement bias task performance in dogs	Observational/quasi-experimental	Examine associations between personality traits and JBT performance
Cockburn et al. (46)	2018	Applied Animal Behavior Science	Evidence of negative affective state in Cavalier King Charles Spaniels with syringomyelia	Observational/quasi-experimental	Examine whether syringomyelia is associated with more negative judgment bias
Piotti et al. (50)	2018	Learning and Behavior	Effect of age on discrimination learning, reversal learning, and cognitive bias in family dogs	Observational/quasi-experimental	Examine age-related differences in learning and cognitive bias
Pogány et al. (51)	2018	Royal Society Open Science	The behavior of overweight dogs shows similarity with personality traits of overweight humans	Observational/quasi-experimental	Examine associations between body condition, behavior and judgment bias
Uccheddu et al. (45)	2018	Dog Behavior	Behavioral and cortisol responses of shelter dogs to a cognitive bias test after olfactory enrichment with essential oils	Experimental	Test whether olfactory enrichment affects JBT performance and cortisol responses
Bunford et al. (23)	2019	Scientific Reports	Individual differences in response to ambiguous stimuli in a modified Go/No-Go paradigm are associated with personality in family dogs	Cross-sectional (mixed design with an experimental component)	Examine individual differences and personality associations in responses to ambiguous stimuli
Duranton and Horowitz (36)	2019	Applied Animal Behavior Science	Let me sniff! Nosework induces positive judgment bias in pet dogs	Experimental	Test whether nosework training induces a more positive judgment bias
Gruen et al. (26)	2019	Journal of Veterinary Behavior	Does the cognitive bias test in dogs depend on spatial learning?	Methodological	Determine whether spatial-learning ability influences spatial JBT performance
Willen et al. (37)	2019	Applied Animal Behavior Science	Enrichment centered on human interaction moderates fear-induced aggression and increases positive expectancy in fearful shelter dogs	Experimental	Test whether human-interaction enrichment influences positive expectancy in shelter dogs
Burani et al. (27)	2020	PLoS ONE	Using judgment bias test in pet and shelter dogs: Methodological and statistical caveats	Methodological	Evaluate methodological and statistical factors affecting interpretation of spatial JBT results
Harvey et al. (47)	2020	Applied Animal Behavior Science	Investigating putative depression-like states in the domestic dog	Observational/quasi-experimental	Examine whether waking inactivity is associated with judgment bias
Hobbs et al. (32)	2020	Scientific Reports	Impact of canine epilepsy on judgement and attention biases	Observational/quasi-experimental	Examine whether idiopathic epilepsy is associated with judgment and attention biases
Vieira de Castro et al. (34)	2020	PLoS ONE	Does training method matter? Evidence for the negative impact of aversive-based methods on companion dog welfare	Observational/quasi-experimental	Compare welfare and judgment bias in dogs exposed to different training methods
Casey et al. (35)	2021	Scientific Reports	Dogs are more pessimistic if their owners use two or more aversive training methods	Observational/quasi-experimental	Examine the association between owners’ use of aversive training methods and judgment bias
Cimarelli et al. (44)	2021	Animal Cognition	Partial rewarding during clicker training does not improve naïve dogs’ learning speed and induces a pessimistic-like affective state	Experimental	Test whether partial versus continuous reinforcement affects learning and subsequent judgment bias
Burani et al. (24)	2022	Animal Cognition	A promising novel judgement bias test to evaluate affective states in dogs	Methodological	Develop and evaluate a modified spatial JBT designed to improve acquisition and discrimination
Chan et al. (38)	2023	Animals	Here Puppy, Chew on This: Short-term provision of toys does not improve welfare in companion dogs	Experimental	Test whether short-term toy provision affects welfare and JBT performance
Wilson et al. (28)	2023	Animal Cognition	The effect of repeated testing on judgement bias in domestic dogs	Methodological	Assess the effect of repeated testing and the test–retest reliability of JBT performance
Krahn et al. (33)	2024	Animal Cognition	Effect of pre-session discrimination training on performance in a judgement bias test in dogs	Experimental	Test whether additional discrimination training influences subsequent JBT performance
Parr-Cortes et al. (31)	2024	Scientific Reports	The odor of an unfamiliar stressed or relaxed person affects dogs’ responses to a cognitive bias test	Experimental	Test whether human stress versus relaxed odor influences JBT responses
Bistre Dabbah et al. (49)	2025	PLoS ONE	An exploratory study of associations between judgement bias, demographic and behavioral characteristics, and detection task performance in medical detection dogs	Observational/quasi-experimental	Examine associations between judgment bias, dog characteristics and medical detection performance
Kinnaird and Wells (41)	2025	Journal of Comparative Psychology	Auditory stimulation and cognitive bias in the domestic dog	Experimental	Test whether different auditory conditions influence JBT performance

Throughout this section, findings are reported as they were observed and analyzed by the original authors. Where an interpretation of a finding is given, it is attributed to the authors of the study concerned; interpretations of our own are reserved for the Discussion.

#### 3.1.1 Risk of bias assessment

Risk-of-bias findings reported in this section refer specifically to the experimental subset assessed with SYRCLE; observational and methodological studies remained part of the systematic review and narrative synthesis but were not included in this design-specific appraisal.

Risk-of-bias ratings for the 15 experimental studies are summarized in Table 6. Most studies were rated as unclear or high risk in domains related to allocation, housing, and blinding. Only Kis et al. (30) and Parr-Cortes et al. (31) received low-risk ratings across the majority of SYRCLE domains, and no study received uniformly low-risk ratings across all domains.

**Table 6:** Risk of bias assessment.

References	A	B	C	D	E	F	G	H	I	J
Experimental studies—assessed with SYRCLE risk of bias tool										
Bunford et al. (23)	NC	Y	NC	NC	N	NC	NC	Y	Y	Y
Burman et al. (21)	NC	Y	NC	Y	N	NC	NC	Y	Y	Y
Chan et al. (38)	NC	NC	NC	N	N	NC	N	NC	Y	N
Cimarelli et al. (44)	NC	Y	NC	Y	N	NC	NC	Y	Y	Y
Duranton and Horowitz (36)	Y	Y	NC	NC	N	NC	NC	Y	Y	Y
Karagiannis et al. (43)	N	N	N	N	N	NC	N	Y	Y	N
Kinnaird and Wells (41)	Y	Y	NC	NC	N	NC	NC	Y	Y	Y
Kis et al. (30)	Y	Y	Y	NC	Y	NC	Y	Y	Y	Y
Müller et al. (39)	NC	Y	NC	NC	N	NC	N	Y	Y	N
Krahn et al. (33)	NC	Y	NC	NC	N	NC	NC	N	Y	N
Parr-Cortes et al. (31)	Y	Y	Y	NC	Y	NC	Y	N	Y	N
Starling et al. (22)	N	N	N	NC	N	NC	NC	N	Y	N
Uccheddu et al. (45)	Y	Y	NC	N	N	NC	NC	Y	Y	N
Walker et al. (40)	N	Y	NC	N	N	NC	N	Y	Y	N
Willen et al. (37)	N	NC	NC	N	N	NC	N	N	Y	N

SYRCLE domains: A: Was the allocation sequence adequately generated and applied? B: Were the groups similar at baseline, or were they adjusted for confounders in the analysis? C: Was the allocation adequately concealed? D: Were the animals randomly housed during the experiment? E: Were the caregivers and/or investigators blinded from knowledge of which intervention each animal received? F: Were animals selected at random for outcome assessment? G: Was the outcome assessor blinded? H: Were incomplete outcome data adequately addressed? I: Are reports of the study free of selective outcome reporting? J: Was the study apparently free of other problems that could result in high risk of bias? Risk of bias ratings: Y, Low risk of bias; N, High risk of bias; NC, Unclear risk of bias (not reported or insufficient information).

Each of the 15 experimental studies was assessed independently by two reviewers across all 10 domains. Inter-rater agreement was 92.0%, with 138 of 150 domain-level judgments concordant (Cohen’s kappa = 0.88); the 12 discrepancies were resolved by consensus.

Domain-level patterns revealed consistent methodological limitations. Adequate sequence generation was documented in 5 of 15 studies (33%), with the remaining studies either lacking adequate randomization or providing insufficient information to confirm it. Allocation concealment was clearly reported in only 2 studies (13%). Random housing was infrequently reported, with only 2 studies receiving a low-risk rating for this domain. Blinding of caregivers or investigators was uncommon, with 13 of 15 studies rated at high risk, while outcome-assessor blinding was clearly documented in only 2 studies (13%); only two studies Kis et al. (30) and Parr-Cortes et al. (31) were double-blinded, that is, blinded on both of these domains—the caregivers or investigators delivering the intervention (SYRCLE domain E) and the assessor scoring the outcome (SYRCLE domain G). Random selection for outcome assessment was not clearly reported in any study. Selective outcome reporting was generally satisfactory, with all 15 studies receiving a low-risk rating for this domain. Incomplete outcome data represented an additional concern in several studies.

Overall, the risk-of-bias assessment indicates that randomization, allocation procedures, blinding, and incomplete outcome data remain important methodological challenges within the experimental canine JBT evidence base.

### 3.2 Synthesized findings

The synthesized findings are presented across five thematic domains: JBT paradigm typology (section 3.2.1), validity and reliability (section 3.2.2), experimental manipulations (section 3.2.3), observational associations (section 3.2.4), and convergent validity (section 3.2.5). A summary table accompanies each subsection, listing the studies synthesized in that subsection.

#### 3.2.1 JBT paradigm typology

Across the 32 included studies, five distinct JBT paradigm types were identified: spatial discrimination (*n* = 28), visual discrimination (*n* = 1), auditory discrimination (*n* = 1), Go/No-Go touchscreen (*n* = 1), and attention bias (*n* = 1, used alongside a spatial JBT). The methodological features of each paradigm type are described below; findings from individual studies are reported in sections 3.2.2–3.2.5.

##### 3.2.1.1 Spatial JBT paradigm (*n* = 28)

The spatial discrimination paradigm, first applied to domestic dogs by Mendl et al. (20), was the dominant approach. In the standard version of this task, dogs are trained to discriminate between two spatial locations: one consistently associated with a positive outcome (e.g., food reward) and one associated with a negative or neutral outcome (e.g., empty bowl or absence of reward). Once dogs reach a predetermined learning criterion, they are presented with one or more ambiguous probe locations positioned at intermediate points between the trained positive and negative locations. The latency to approach these ambiguous probes serves as the primary outcome measure, with shorter latencies interpreted as reflecting a more optimistic (positive) affective state, and longer latencies as reflecting a more pessimistic (negative) affective state. Across studies, the total number of tested locations ranged from three to five, and the number of ambiguous probes from one to three, with five-location paradigms (positive, near-positive, middle, near-negative, negative) becoming increasingly common. Burani et al. (24) introduced a five-corridor variant with greater physical separation between locations to improve spatial discrimination (see section 3.2.2).

##### 3.2.1.2 Visual JBT paradigm (*n* = 1)

Burman et al. (21) developed a visual discrimination paradigm using grayscale reference cards. Dogs were trained to discriminate between two reference stimuli (a light and a dark card) associated with different outcomes, then tested with an intermediate gray card. This paradigm removes the spatial component entirely, relying instead on luminance discrimination and potentially engaging different cognitive processes than spatial tasks.

##### 3.2.1.3 Auditory JBT paradigm (*n* = 1)

Starling et al. (22) pioneered an automated auditory paradigm in which dogs were trained to associate two tone frequencies with positive and negative outcomes, then tested with intermediate frequencies. The study also introduced Cox proportional hazard models and tipping point analysis as novel statistical approaches for JBT data, expanding the methodological repertoire beyond the latency-based analyses typical of spatial designs.

##### 3.2.1.4 Go/No-Go touchscreen paradigm (*n* = 1)

Bunford et al. (23) developed a Go/No-Go paradigm using colored geometric shapes presented on a touchscreen. Dogs were trained to touch (“go”) for one stimulus and withhold responding (“no-go”) for another, with ambiguous intermediate stimuli presented during testing. This paradigm offers the conceptual advantage of an active choice measure (go vs. no-go) over the approach-latency measure of spatial designs, though it requires substantially more training sessions (18+) and has only been employed in a single study to date.

##### 3.2.1.5 Attention bias paradigm (*n* = 1)

Hobbs et al. (32) incorporated an attention bias measure, in which dogs’ latency to approach a positively-valenced stimulus (food) is measured following exposure to a threatening stimulus. Unlike standard JBT paradigms that require discrimination training, attention bias tasks assess threat-related attentional processing more directly and may capture complementary aspects of affective state.

#### 3.2.2 Validity and reliability of JBT measures

Five studies specifically addressed the validity and reliability of JBT measures in domestic dogs, identifying critical methodological concerns that have implications for interpreting findings across the entire corpus.

The most consequential validity challenge involves spatial working memory as a confounding variable. Gruen et al. (26) administered both a standard five-location spatial JBT and a delayed non-match to position (DNMP) spatial working memory task to 16 laboratory-housed Labrador Retrievers. Dogs requiring fewer trials to acquire the DNMP task showed significantly shorter JBT latencies to the near-positive (*r*^2^ = 0.56, *p* = 0.001) and near-negative probes (*r*^2^ = 0.39, *p* = 0.013). Only 9 of 16 dogs acquired the DNMP task, further highlighting individual variability.

Burani et al. (27) conducted a systematic methodological analysis in 91 dogs (51 shelter, 40 pet), revealing three critical issues. First, shelter dogs appeared more pessimistic than pet dogs when raw latency data were analyzed, but this difference disappeared entirely when latencies were adjusted for individual baseline running speed; the authors concluded that raw trial-level latencies are preferable to averaged adjusted scores, and that adjusted scores should be interpreted with caution as indices of optimism or pessimism. Second, a substantial proportion of dogs failed to reach the training criterion. Third, dogs showed poor discrimination between adjacent spatial locations.

Burani et al. (24) directly addressed these limitations by developing an improved five-corridor spatial paradigm, tested with 49 pet dogs. The redesigned apparatus featured greater physical separation between corridor locations and standardized training procedures. Results were substantially improved: 98% of dogs reached the learning criterion, compared to 69–78% in typical implementations. Dogs discriminated reliably between all adjacent location pairs, and the authors reported behavioral indicators (tail wagging at the positive location, hesitation at the negative location) consistent with learning of the location-outcome contingencies.

Wilson et al. (28) provided direct evidence of temporal instability by testing 15 dogs across five sessions without any intervening affective manipulation. Dogs became progressively and significantly slower to approach ambiguous locations (*p* < 0.05), non-approach responses increased, and intraclass correlation coefficients (ICC) for test–retest reliability were consistently poor (0.009–0.333).

Krahn et al. (33) investigated whether additional discrimination training prior to JBT sessions would artificially alter optimism scores in a quasi-experimental design with 16 dogs (8 experimental, 8 yoked controls). Pre-session training did not significantly alter JBT performance (F3, 9 = 0.24, *p* = 0.86), nor did it affect the proportion of approaches to the ambiguous locations (F3, 5 = 0.13, *p* = 0.94).

#### 3.2.3 Manipulation- and intervention-related evidence

Fifteen studies were synthesized in relation to manipulation- or intervention-related differences in JBT performance. Their designs ranged from randomized and within-subject experiments to quasi-experimental interventions and observational comparisons of previous training experience. Accordingly, the evidence is described according to study design rather than treated uniformly as experimental. Rather than relying on a binary vote-counting summary, we summarize the evidence here by manipulation or intervention domain. The number of studies in each domain was small (two pharmacological, three training-related, four enrichment, two social, and one each for olfactory and auditory manipulations), with two additional studies providing counterintuitive or methodologically informative findings. The grouping is therefore descriptive and is not intended to support inferences about which classes of manipulation the JBT is most sensitive to.

Studies in the pharmacological and training-related domains most consistently reported shifts in the predicted direction. However, the training-related evidence included observational or quasi-experimental comparisons of previous training experience as well as a randomized manipulation of reinforcement schedule, and these findings therefore differ in the strength of causal inference they support. Standardized effect sizes were, however, reported for only two of these studies, and only one is large and directly interpretable [(30): Cohen’s *d* = 2.16]. The remaining studies reported test statistics and *p*-values only [(34): *p* < 0.05; (35): Z = −2.38, *p* = 0.017]. A Z-score expresses how far an observed value lies from the value expected under the null hypothesis, and a *p*-value the probability of an observation at least as extreme if the null hypothesis is true; neither is a measure of effect magnitude, and neither should be read as one. Enrichment-based manipulations were directionally consistent but more variable, with clear effects in some contexts [(36): *d* = 0.64; (37) in fearful dogs] and null effects in others [(38): *p* = 0.905].

Olfactory, social, and auditory manipulations were mixed: Parr-Cortes (31) detected near-negative latency shifts to human stress odor, while Müller et al. (39), Walker (40), and Kinnaird and Wells (41) reported null JBT effects of owner absence, conspecific separation, and auditory stimulation, respectively. Two studies produced counterintuitive or methodologically informative findings (Burman (21): post-reward pessimism in a visual paradigm; Starling (22): declining response rates across sessions). These patterns are detailed below by manipulation type.

##### 3.2.3.1 Pharmacological interventions

Kis et al. (30) conducted a between-subjects 2 × 2 factorial study (*N* = 64), administering 12 IU intranasal oxytocin versus placebo in communicative versus non-communicative social contexts. Oxytocin produced a significant main effect on optimistic bias [*F* (1, 60) = 38.818, *p* < 0.001], with very large effect sizes in the communicative context (*d* = 2.163) and large effects in the non-communicative context (*d* = 0.965). Oxytocin simultaneously decreased heart rate [Cohen’s *d* = 0.944 for the within-subject reduction in heart rate; paired *t*-test, *t*(9) = 2.810, *p* = 0.020, *n* = 10] and increased heart rate variability (*d* = 1.400). Despite the growing evidence that variation in the canine oxytocinergic system is associated with measurable behavioral differences (42), the *d* = 2.163 estimate should be interpreted with caution: with the 64 dogs distributed across four cells (~16 per cell), sampling error is substantial and the corresponding 95% confidence interval is wide. Effect estimates of this magnitude in small samples are vulnerable to upward bias, and replication in larger samples is warranted before this value is treated as a stable benchmark.

Karagiannis et al. (43) examined combined fluoxetine and behavior modification in a pre-post design with 5 dogs with separation-related problems (SRP) and 7 controls. SRP dogs showed significantly more pessimistic bias at baseline (*p* < 0.05), and after 6 weeks of treatment, their bias normalized to control levels (*p* > 0.05), with clinical improvement correlating with bias change.

##### 3.2.3.2 Training methodology

Two observational/quasi-experimental studies examined associations between previous training methods and JBT performance. Vieira de Castro et al. (34) compared 92 dogs classified by training method (42 reward-based, 22 mixed, 28 aversive) through objective video coding of training classes. Dogs in the aversive group showed significantly more stress behaviors during training (*p* < 0.001), higher post-training salivary cortisol (*p* < 0.001), and more pessimistic cognitive bias (*p* < 0.05), with the mixed group showing intermediate values.

Casey et al. (35) extended these findings with a matched-pair case–control design (*N* = 100; 50 pairs), demonstrating that dogs trained with two or more aversive methods were significantly slower to approach the middle (*Z* = −2.380, *p* = 0.017) and near-positive (*Z* = −2.447, *p* = 0.014) ambiguous locations.

By contrast, Cimarelli et al. (44) explored reinforcement schedules in a randomized controlled trial comparing partial (60%) to continuous (100%) reward during clicker training. While learning speed did not differ, dogs in the partial reinforcement condition showed significantly more pessimistic bias (*p* < 0.05), and emotional reactivity was associated with more pessimistic responding.

##### 3.2.3.3 Environmental enrichment

Duranton and Horowitz (36) conducted a randomized controlled trial with 20 pet dogs, comparing 2 weeks of nosework training (*n* = 10) to heelwork training as an active control (*n* = 10). The nosework group showed significantly shorter latencies to the ambiguous location post-intervention (*p* < 0.01, *d* = 0.64), with 99% inter-rater reliability.

Uccheddu et al. (45), in one of the largest studies by sample size (*N* = 110), tested nine individual essential oils plus a blend on shelter dogs in a between-subjects RCT. Only the oil blend significantly reduced latency to the ambiguous location (*p* = 0.039); lavender reduced cortisol (*p* = 0.039) but did not alter cognitive bias.

Willen et al. (37) examined daily human interaction enrichment in shelter dogs across two experiments (Exp 1: *N* = 60 dogs; Exp 2: *N* = 64 dogs), stratified by fearfulness. In fearful dogs, enrichment dramatically improved SAFER behavioral assessment pass rates (94% vs. 13%, *p* < 0.001) and reduced latencies to ambiguous locations (*p* < 0.01). However, in non-fearful dogs, a significant effect in the opposite direction was observed (*p* < 0.05).

Chan et al. (38) tested toy provision (simple vs. complex toys vs. control) in 47 companion dogs over 12 days but found no significant differences in cognitive bias, eating duration, or activity levels (the latter recorded with a FitBark, a collar-mounted accelerometer-based activity monitor).

##### 3.2.3.4 Olfactory, social, and auditory manipulations

Parr-Cortes et al. (31) conducted a within-subjects, double-blinded study (*N* = 18) exposing dogs to human stress odor, relaxed odor, or no odor across three sessions. Dogs showed more pessimistic responses to stress odor, particularly at the near-negative location, with slower approach latencies and lower approach rates.

Müller et al. (39) tested 24 pet dogs in a within-subjects design, comparing JBT performance under owner-present and owner-absent conditions. In the linear mixed-model analysis, owner presence did not significantly affect adjusted latency scores to ambiguous locations [*F* (1, 537) = 0.04, *p* = 0.84], whereas test day had a significant effect, with longer latencies recorded on the second test day [*F* (1, 537) = 25.5, *p* < 0.001]. No significant differences in latency scores were detected among breed groups [*F* (3, 19) = 0.25, *p* = 0.86].

Walker et al. (40) investigated conspecific separation in 12 pair-housed shelter dogs. While separation significantly increased activity behaviors (running: *p* = 0.02; circling: *p* = 0.006), decreased play (*p* = 0.01), and elevated secretory immunoglobulin A (sIgA; *p* = 0.02), no significant change in cognitive bias was detected (*p* = 0.33).

Kinnaird and Wells (41) tested 45 dogs across three auditory conditions (classical music, audiobook, silence) in a between-subjects design. No significant effects of auditory condition on JBT latencies were found; however, the expected location gradient was confirmed (*p* < 0.001), and latencies increased across trials. Neutered dogs also required more training trials (*p* = 0.04).

##### 3.2.3.5 Counterintuitive and methodological findings

Starling et al. (22), employing an automated auditory paradigm with 40 dogs, observed that dogs became less likely to respond across successive test sessions.

#### 3.2.4 Observational studies: associations with welfare indicators and individual characteristics

Twelve studies employed observational or quasi-experimental designs to examine naturally occurring associations between JBT performance and individual characteristics, without deliberate manipulation of affective states.

##### 3.2.4.1 Clinical and medical conditions

Mendl et al. (20), in the foundational study of this corpus, compared 24 shelter dogs [12 with separation-related behavior (SRB) and 12 controls] on a spatial JBT with two trained locations and three ambiguous probes. SRB dogs responded significantly more pessimistically, with longer approach latencies, and SRB frequency correlated positively with pessimistic bias.

Cockburn et al. (46) assessed 21 Cavalier King Charles Spaniels, comparing 8 dogs with MRI-confirmed syringomyelia (SM) to 13 controls. SM dogs showed significantly more negative judgment at ambiguous locations (*p* < 0.05), and critically, concurrent analgesic medication did not reverse this bias. A paradoxical positive correlation between scratching score and optimistic bias was also reported.

Hobbs et al. (32) compared 33 dogs with idiopathic epilepsy (IE) to 35 matched controls (Border Collies and Labradors) using both spatial JBT and a novel attention bias task. No significant JBT difference was detected [*F* (1, 102) = 0.20, *p* = 0.658], but IE dogs were significantly more likely to fail the learning criterion (*p* = 0.019). The attention bias task detected a significant effect: IE dogs showed increased latency to approach a positive stimulus following an aversive sound (*p* = 0.031).

Harvey et al. (47) tested 20 shelter dogs (Royal Society for the Prevention of Cruelty to Animals) to examine whether waking inactivity (a proposed analogue of behavioral depression) correlated with JBT. Most dogs showed predominantly optimistic bias, and no significant association was found. Stray dogs learned the task faster than seized or relinquished dogs (*p* = 0.037).

##### 3.2.4.2 Personality and temperament

Barnard et al. (48) assessed 31 pet dogs using a five-location spatial paradigm alongside two validated personality instruments (DMA and C-BARQ). Higher sociability predicted shorter near-negative latencies (*p* = 0.03), higher separation-related problem scores predicted longer near-positive latencies (*p* = 0.002), and dog-directed fear was associated with longer latencies to both ambiguous locations (*p* = 0.0004).

Bunford et al. (23), using the Go/No-Go touchscreen paradigm with 29 family dogs, found that higher conscientiousness and extraversion predicted more “go” (optimistic) responses (partial eta-squared = 0.19 and 0.14 respectively). A replication analysis confirmed the extraversion-bias association, and no effect of inattention was detected.

Bistre Dabbah et al. (49) examined 58 medical detection dogs (39 prospective/trainee dogs and 19 working dogs at enrolment; data were subsequently available for 27 trained dogs) using a spatial paradigm with three ambiguous positions. More optimistic dogs showed significantly higher task ability scores (*p* = 0.002), while more pessimistic dogs demonstrated higher detection specificity (*p* = 0.021), meaning fewer false alarms. Older dogs showed better overall detection performance (*p* < 0.001).

##### 3.2.4.3 Demographic and physical characteristics

Piotti et al. (50) conducted one of the largest studies in the corpus (*N* = 107 family dogs), comparing young (2.5–6.5 years) and older (8–14.5 years) dogs using spatial and feature-based paradigms. Young dogs learned the discrimination significantly faster, and most older dogs failed to complete reversal learning. No cognitive bias difference was detected between age groups, which the authors attributed to narrower response contrasts in older dogs.

Pogány et al. (51) compared overweight and normal-weight dogs (*N* = 91, five breeds) on a three-location paradigm. Overweight dogs approached the ambiguous location significantly more slowly (*p* = 0.026), while working dogs were less food-motivated overall.

Wells et al. (52) tested 30 pet dogs on a five-location paradigm and found that motor laterality significantly influenced cognitive bias: left-pawed dogs approached ambiguous positions more slowly, with a strong correlation between the handedness index and near-positive probe latency (*r* = 0.66, *p* < 0.001). Right-pawed and ambilateral dogs showed the expected latency gradient.

##### 3.2.4.4 Welfare-environment associations

Titulaer et al. (53) compared 53 Dogs Trust (a UK dog-rehoming charity) shelter dogs in short-term and long-term kenneled groups, assessing cognitive bias, urinary cortisol:creatinine ratios, behavior, and staff welfare questionnaires. No significant differences emerged on any measure, despite long-term dogs showing more resting behavior.

Owczarczak-Garstecka and Burman (54) observed 15 shelter dogs over 5 days, assessing sleep and rest patterns, repetitive behaviors, staff welfare ratings, and JBT performance. Higher daytime resting was significantly associated with more positive JBT bias, less repetitive behavior, and higher staff-rated relaxation.

#### 3.2.5 Convergent validity with independent welfare measures

An important dimension of JBT validity is the extent to which cognitive bias measures converge with independent welfare indicators. Eighteen studies provided data relevant to this question. The overall pattern reveals partial convergence: several studies demonstrate coherent alignment between JBT and other welfare measures, while others reveal dissociations that raise important interpretive questions.

##### 3.2.5.1 Physiological convergence

The strongest physiological convergence was demonstrated by Kis et al. (30), whose oxytocin manipulation simultaneously modulated autonomic (heart rate, HRV) and cognitive measures in a coherent pattern.

Vieira de Castro et al. (34) similarly found alignment across cortisol, stress behaviors, and pessimistic bias. However, dissociations also emerged: Uccheddu et al. (45) found that lavender reduced cortisol without altering bias, while a different oil blend (the multi-component blend tested in that study, as distinct from the single essential oils, including lavender) altered bias without comparable cortisol effects.

Walker et al. (40) reported robust immune and behavioral responses to separation but no corresponding change in cognitive bias.

##### 3.2.5.2 Behavioral convergence

Willen et al. (37) demonstrated strong convergence between SAFER behavioral assessment improvements and optimistic bias shifts in fearful shelter dogs. Mendl et al. (20), Casey et al. (35), and Vieira de Castro et al. (34) showed alignment between validated clinical or behavioral assessments and bias direction. Owczarczak-Garstecka and Burman (54) provided multi-indicator convergence in shelter dogs (rest, repetitive behavior, staff ratings, JBT), while Karagiannis et al. (43) showed clinical improvement paralleling bias normalization during treatment.

Duranton and Horowitz (36) found convergence between nosework-induced optimistic bias and reduced stress behaviors. By contrast, Titulaer et al. (53) found no convergence across any modality, and Harvey et al. (47) found no association between waking inactivity and bias, though ceiling effects may have limited sensitivity.

##### 3.2.5.3 Neurobiological and individual difference correlates

Wells et al. (52) found a strong association between cerebral lateralization and cognitive bias (*r* = 0.66, *p* < 0.001). Barnard et al. (48) and Bunford et al. (23) reported associations between personality measures and cognitive bias. Cockburn et al. (46) showed that MRI-confirmed syringomyelia predicted pessimistic bias, though other physiological correlates showed partial convergence. Hobbs et al. (32) provided an informative case of divergent validity: spatial JBT and attention bias yielded different results within the same epilepsy population. Bistre Dabbah et al. (49) provided a novel perspective linking bias to functional working outcomes (optimism = task ability; pessimism = detection specificity).

##### 3.2.5.4 Confounds affecting interpretation

Gruen et al. (26) demonstrated that spatial working memory confounds JBT, meaning apparent convergence between JBT and other measures may be mediated by cognitive ability rather than affective state.

Burman et al. (21) showed that motivational state can override expected affective influences, complicating interpretation of studies using food reward as both JBT reinforcer and mood induction.

## 4 Discussion

### 4.1 Summary of main findings

Across the 32 included studies, the JBT in dogs is operationally feasible but methodologically fragile. With respect to validity and reliability (RQ1), the dominant spatial paradigm is acquired in most samples but is confounded by spatial-learning ability (26), sensitive to analytic choices about raw versus adjusted latencies (27) and shows poor test–retest reliability with progressive pessimistic drift across sessions (28). With respect to RQ2, the studies synthesized in Table 3 showed the clearest shifts in the predicted direction in the pharmacological and training-related domains (30, 34, 35, 43, 44), with weaker but generally directionally consistent effects for enrichment (36–38, 45) and mixed results for olfactory, social, and auditory manipulations (31, 39–41). However, the training-related evidence includes observational and quasi-experimental comparisons (34, 35), and these findings should therefore not be interpreted as uniformly causal. This domain-level pattern should not be read as evidence that the JBT is differentially sensitive to different classes of manipulation. Each domain rests on very few studies (two pharmacological, three training-method, four enrichment, two social, and one each for olfactory and auditory manipulations), and those studies differ from one another in sample size, blinding, latency operationalization and analytic approach as much as they differ in the intervention applied. With an evidence base of this size it is not possible to separate the effect of the type of manipulation from the effect of how the individual studies were conducted. The single very large effect [(30), *d* = 2.16] is informative but should be treated cautiously given the small per-cell sample (~16 dogs) and the corresponding wide confidence interval.

Observational data link pessimistic bias to clinical pain (46), aversive training history (34, 35), personality (48), laterality (52), and body condition (51), while age (50), kenneling duration (53), and epilepsy (32) show no JBT-level effect. Convergence with independent welfare indicators is partial: cortisol, behavior, and physiology align with JBT in some studies (30, 34, 37, 54) but dissociate in others (40, 45), suggesting that the JBT and physiological/behavioral measures may operate on different timescales or capture related but non-identical constructs.

### 4.2 Methodological considerations and sources of heterogeneity

It is useful to frame the methodological landscape of the canine JBT in classical psychometric terms. Construct validity, whether the JBT measures the intended affective construct rather than an alternative such as spatial-learning ability or decision uncertainty, is challenged on two fronts. First, Gruen et al. (26) documented a substantial spatial-memory confound. Second, latency-based outcomes do not, on their own, discriminate between affective and non-affective mechanistic accounts. Convergent validity, whether JBT scores covary with independent indicators of the same construct, is partially supported (30, 34, 37, 54) but not universal.

The dissociations between JBT, cortisol, and behavioral measures observed by Walker et al. (40) and Uccheddu et al. (45) show that the JBT does not co-move with conventional welfare proxies in all contexts. Discriminant validity (whether the JBT differentiates affective state from related but distinct constructs such as motivational or attentional processes) remains essentially untested in the canine literature. Test–retest reliability, the most basic psychometric prerequisite for longitudinal welfare monitoring, is also poor: Wilson et al. (28) reported intraclass correlation coefficients of 0.009–0.333 across five sessions, with a systematic drift toward pessimistic responding. Together, these psychometric weaknesses indicate that the canine JBT, as currently deployed, does not yet meet conventional thresholds for a stable clinical or applied measurement instrument (Table 7).

**Table 7:** Convergent-validity matrix for studies synthesized in section 3.2.5.

References	Welfare indicators measured alongside JBT	Convergence with JBT
Kis et al. (30)	Heart rate, heart rate variability	Yes (full convergence)
Vieira de Castro et al. (34)	Salivary cortisol, training stress behaviors	Yes (full convergence)
Casey et al. (35)	Owner-reported training methods	Yes (partial)
Karagiannis et al. (43)	Clinical SRP severity, behavior modification outcome	Yes (full convergence)
Duranton and Horowitz (36)	Stress behaviors during training	Yes (partial)
Willen et al. (37)	SAFER behavioral assessment, fear behaviors	Yes (in fearful dogs only)
Owczarczak-Garstecka and Burman (54)	Sleep/rest patterns, repetitive behaviors, staff welfare ratings	Yes (multi-indicator)
Mendl et al. (20)	Separation-related behavior score	Yes (partial)
Wells et al. (52)	Paw preference (motor laterality)	Yes (theoretical convergence; *r* = 0.66)
Barnard et al. (48)	Personality (DMA, C-BARQ)	Yes (partial)
Bunford et al. (23)	Personality (Five-Factor instruments)	Yes (partial)
Cockburn et al. (46)	MRI-confirmed syringomyelia, scratching score	Yes (partial; paradoxical scratching pattern)
Bistre Dabbah et al. (49)	Detection-task ability, specificity, age	Yes (functional outcome convergence)
Uccheddu et al. (45)	Salivary cortisol	Partial/dissociation (different oils → different markers)
Walker et al. (40)	Activity behaviors, sIgA	No (immune/behavioral change without bias change)
Titulaer et al. (53)	Cortisol:creatinine ratio, behavior, staff ratings	No (no convergence across modalities)
Harvey et al. (47)	Waking inactivity (depression analogue)	No (ceiling effects in JBT)
Hobbs et al. (32)	Attention bias, epilepsy status	Divergent (JBT and attention-bias measures dissociate)
Gruen et al. (26)	Spatial working memory (DNMP)	Confound (apparent convergence mediated by spatial ability)
Burman et al. (21)	Food reward/motivational state	Confound (motivation overrides expected affective effect)

C-BARQ, Canine Behavioral Assessment and Research Questionnaire; DMA, Dog Mentality Assessment; DNMP, delayed non-match to position task; JBT, judgment bias task; MRI, magnetic resonance imaging; SAFER, Safety Assessment for Evaluating Rehoming; sIgA, secretory immunoglobulin A; SRP, separation-related problems.

Several observations reported in Section 3 bear directly on this psychometric appraisal, and we set out our reading of them here rather than in the Results. The association between spatial-learning ability and probe latencies reported by Gruen et al. (26) is, in our view, most economically explained by dogs with better spatial learning having more rapidly learned which locations are never rewarded, so that shorter latencies index learning rather than positive affect. The progressive slowing across sessions reported by Wilson et al. (28), in the absence of any affective manipulation, is likewise most parsimoniously explained by dogs learning that ambiguous probes are unreinforced, which drives responses in a pessimistic direction independently of affective state; the null effect of pre-session discrimination training reported by Krahn et al. (33) suggests that this drift arises from exposure to the unreinforced probes themselves rather than from the discrimination-training component. The weak discrimination between adjacent locations reported by Burani et al. (27) raises the question of whether the five-location paradigm measures a graded response at all, or effectively a dichotomous positive-versus-negative classification. Finally, the higher failure rate on the learning criterion among dogs with idiopathic epilepsy reported by Hobbs et al. (32) illustrates a more general problem: because dogs that fail training are excluded, samples are selected for cognitive ability, and this selection acts most strongly in exactly the clinical populations in which the JBT would be most useful.

The field’s methodological heterogeneity substantially limits comparative inference. Five distinct paradigm types were identified, with spatial dominating. This paradigm diversity reflects adaptive innovation but creates a practical impediment to meta-analysis and synthesis. The spatial paradigm itself varies considerably: total number of tested locations (3–5), training protocols, latency measurement procedures, and ambiguous stimulus definitions differ markedly across studies. Burani et al. (27) demonstrated that these variations are not merely cosmetic. Raw versus adjusted latencies yielded different conclusions, and adjacent compartment discrimination was substantially weaker than predicted, suggesting that dogs may respond mainly to how far the bowl is from the rewarded location, rather than to a learned association between each specific location and the outcome it predicts.

Spatial memory confounding is a critical but frequently unaddressed confound. Gruen et al. (26) provided strong evidence (*r*^2^ = 0.56) that spatial cognition significantly predicts JBT performance. This raises a fundamental question: to what extent does the JBT measure affective state versus spatial-cognitive abilities? Studies have attempted to control for this [e.g., Burani et al. (27) used adjusted latencies], but standardized methods for detecting and correcting spatial confounds remain absent. Relatedly, the assumption that increases in latency over the course of reinforcement directly reflect affective state warrants scrutiny: alternative mechanisms including habit formation, motor learning, and decision processing uncertainty may generate identical latency patterns.

Training success variability (50–98%) indicates inconsistent baseline establishment across studies. This variability likely reflects differences in dog population characteristics (age, experience, breed, motivation), trainer expertise, and criterion definitions, none of which are typically adequately documented or controlled. This is particularly problematic because incomplete training may introduce ceiling or floor effects that confound subsequent bias measurement. Furthermore, repeated-testing effects documented by Wilson et al. (28), together with the longer latencies observed on the second test day by Müller et al. (39), suggest that JBT performance may change across repeated sessions independently of the experimental manipulation, complicating the interpretation of temporally proximal repeated-measures designs.

Our risk-of-bias assessment showed that most experimental studies lacked randomization and blinding. Only Kis et al. (30) (double-blind, between-subjects) and Parr-Cortes et al. (31) (double-blind) achieved strong methodological ratings. This suggests substantial potential for both unintentional experimenter bias and systematic confounding by unmeasured variables within the experimental evidence base. Pre-registered protocols are absent from this field. A single JBT study typically yields several outcomes that can each be tested separately: two or three ambiguous locations, latency and approach/no-approach measures, raw and rescaled latency scores, and repeated test sessions. The scope for reporting only the subset of these that reaches significance is therefore considerable.

Accordingly, the SYRCLE findings inform the interpretation of evidence from experimental manipulation studies, for which randomization, allocation procedures, and blinding emerged as important methodological challenges, whereas conclusions derived from observational and methodological studies are discussed separately according to their respective designs and evidential roles.

### 4.3 Comparison with the broader cognitive bias literature in other species

The JBT protocol has been adapted from research in non-human primates and rodents, where it has demonstrated effects of affective state manipulations, albeit with similar methodological limitations.

In rats, the foundational paradigm of Harding et al. (12) showed that unpredictable housing produced pessimistic interpretations of ambiguous auditory cues, and subsequent research on rodents has built a relatively mature body of evidence on construct validity when multimodal outcome measures are employed (19).

Sheep research has demonstrated bidirectional modulation of judgment bias: release from restraint generates an optimistic shift (13), whereas serotonergic depletion induces a pessimistic shift (14), providing some of the clearest pharmacological and contextual control evidence in any non-human species.

Pig studies have shown that environmental enrichment induces optimistic biases (15), mirroring the directionality of canine enrichment findings (36, 37), and horse studies have linked sub-optimal social, spatial, and feeding conditions to pessimistic responses (16).

Even more taxonomically distant work in zebrafish has shown that the cognitive bias paradigm can detect both positive and negative affective states (17) and that physical environmental enrichment buffers stress responses to novel contexts (18).

What can canine literature learn from these parallel bodies of work? First, the studies on sheep, pig, and zebrafish demonstrate that relatively simple, well-controlled manipulations (restraint release, enrichment, pharmacological agents) can produce reliable affective shifts when paradigms are kept consistent within a research program. The canine field would benefit from comparable within-laboratory replication efforts rather than continued paradigm proliferation.

Second, the cross-species literature shows that multimodal outcome sets, combining behavioral, physiological, and cognitive indicators, substantially strengthen causal inference (12–19). The canine corpus reviewed here applies this principle inconsistently, although with notable exceptions (30, 34, 54).

Third, comparable methodological critiques apply across species: task paradigm heterogeneity, learning and memory confounds, and variability in training adequacy have all been documented in rodent and ungulate literature as well. Solutions developed in one species, such as Cox proportional hazard modeling pioneered for canine auditory paradigms (22), or within-laboratory paradigm standardization in sheep and pig research, should therefore be more actively imported across the cognitive-bias field.

A key difference in the canine literature is the particularly strong emphasis on personality and trait individual differences as predictors of JBT performance. While trait anxiety predicts cognitive bias in other species, the canine studies provide more extensive documentation of personality-bias associations (48). This may reflect both the greater accessibility of personality assessment measures in applied dog populations and the reality that dogs, being domesticated, possess diverse selectively-bred phenotypes unlike most animal models. This raises an important theoretical consideration: to what extent do personality traits and acute affective states independently influence cognitive bias, and do they interact? Most canine JBT studies have not adequately dissociated these constructs.

The broader cognitive bias literature increasingly recognizes that stimulus ambiguity and decision uncertainty, rather than pure affective state, may drive many observed biases. The canine JBT literature has given limited attention to this mechanistic possibility. The counterintuitive post-reward pessimism reported by Burman et al. (21) may reflect increased decision uncertainty following reward acquisition (uncertainty about whether the rewarded compartment will be consistently rewarded) rather than valence-based affective state. This mechanistic ambiguity, whether JBT reflects affective state or decision uncertainty, substantially limits theoretical interpretation.

### 4.4 Implications for animal welfare science and practice

The partial validity and reliability of JBTs in dogs limits their immediate utility as field measures for welfare assessment, though the construct does show promise in controlled research settings. The finding that aversive training produces both pessimistic bias shifts and elevated cortisol (34) provides valuable evidence that training methods robustly influence measurable affective and cognitive outcomes. Similarly, the demonstration that enrichment (nosework training, environmental enrichment) produces measurable bias shifts (36, 37) supports the welfare relevance of such interventions, though the mechanisms remain unclear.

We therefore recommend that practitioners interpret JBT results cautiously rather than as direct measures of individual welfare. The methodological limitations and null findings in several expectancy studies suggest that JBT performance is influenced by multiple factors beyond acute affective state. Two of the null findings reported in Section 3 illustrate this. The absence of any effect of toy provision reported by Chan et al. (38) is consistent with pet dogs in already-enriched home environments being close to a ceiling on the measure, and the absence of a JBT change after conspecific separation reported by Walker et al. (40), despite clear immune and behavioral responses, is consistent with the JBT and physiological indicators responding on different timescales. Neither interpretation is testable with the data reported in those studies. The dissociation between JBT results and other affective indicators (e.g., cortisol, immune function) reported in several studies suggests that reliance on JBT alone may provide an incomplete welfare picture. It should be noted, however, that cortisol is not invariably a reliable marker of stress: when the onset of a stressor is predictable for the individual, the cortisol response may be attenuated or absent. A dissociation between cognitive bias and cortisol therefore does not by itself indicate that either measure is invalid. Multi-method approaches combining JBT with behavioral observation, physiological markers, and health indicators remain advisable.

The demonstration that attention bias measures may diverge from JBT measures (32) suggests that different cognitive bias paradigms may assess distinct constructs and that researchers should not assume interchangeability. This finding has practical implications: if different bias measures assess different aspects of affective processing, then welfare assessment may require multiple complementary measures rather than a single cognitive bias task.

### 4.5 Recommendations for the conduct and reporting of judgment bias studies

Several priority areas for methodological improvement have emerged from this review. First, standardization of spatial paradigm protocols is urgently needed. This should include agreed-upon compartment configurations, training criteria, latency measurement procedures, and procedures for detecting and controlling spatial memory confounds. Pre-specification of analysis protocols and registration of study protocols (e.g., through OSF) would reduce selective reporting risk.

Second, studies should explicitly measure and report potential spatial memory confounds and employ statistical controls where appropriate. If spatial cognition contributes substantially to JBT performance, then either controlling for this variance (via ANCOVA with spatial cognition measures) or employing non-spatial paradigms (visual, auditory, Go/No-Go) may improve measurement validity. The visual (*n* = 1) and auditory (*n* = 1) paradigms identified in this review warrant expanded investigation as potentially less confounded alternatives, despite their current limited evidence base.

### 4.6 Priorities for future research

First, investigations into the mechanisms underlying observed cognitive biases are needed. Does JBT primarily reflect affective state (valence, arousal), or does it measure decision uncertainty, habit formation, or motor learning effects? Studies employing mechanistic dissociations, for example, manipulations predicted to influence affective state but not decision uncertainty, or vice versa, would illuminate this question. The counterintuitive post-reward pessimism documented by Burman et al. (21) warrants systematic investigation to determine whether this reflects genuine emotional effects or an alternative mechanism.

Second, test–retest reliability should be systematically investigated across representative dog populations, with explicit documentation of learning effects and temporal stability. Designs employing retest intervals of varying lengths (1 week, 1 month, 3 months) would establish the temporal scope of reliable measurement and inform the feasibility of longitudinal designs.

Third, developmental applications of the JBT warrant further investigation. A study published after completion of the literature search for the present review demonstrated that a spatial JBT is feasible in puppies at 60 days of age, with puppies showing graded responses to positive, ambiguous, and negative locations (55). This extends the potential application of the paradigm to early canine development and provides a basis for longitudinal studies examining whether early responses to ambiguity remain stable across development or are modified by subsequent environmental and social experience. In particular, assessment before and after adoption could help distinguish relatively stable individual differences from changes associated with subsequent management, training, and owner–dog interactions.

Fourth, convergent validity studies employing multimodal outcome measures, combining JBT, physiological indicators (cortisol, heart rate variability, immune function), behavioral observations, and trait measures, should be expanded. Dissociations between measures require explanation: either different measures assess different constructs, or measurement error in one or more methods obscures true associations.

Fifth, more studies examining experimental manipulations with clear affective predictions are needed, particularly focused on understanding why some manipulations (separation, toy provision, auditory paradigms) have failed to produce predicted effects. Two explanations must be distinguished here: the manipulation may have failed to change the dog’s affective state at all, or it may have changed the affective state without that change being detectable in JBT performance. Separating them requires studies that verify the affective impact of the manipulation with independent behavioral and physiological measures, rather than inferring it from the manipulation itself.

Sixth, individual and social moderators of JBT performance should be investigated more systematically. Personality and acute affective state may independently influence bias, interact with one another, or confound the apparent relationship between experimental manipulations and JBT performance. Explicit within-study examination of personality as a moderator would therefore advance understanding. Related factors may also be relevant: Müller et al. (39) found no significant differences among breed groups and no effect of brief owner absence; however, their study did not directly test working status, breed-related functional selection, or owner–dog relationship quality. These factors therefore warrant targeted investigation as potential moderators of JBT performance.

Seventh, a credible pathway toward clinical and applied translation should be defined. Most of the studies reviewed here were not designed as diagnostic instruments; they tested whether group-level differences in affective state could be detected. For the JBT to support individual welfare or clinical decisions, for example, screening shelter dogs for chronic distress, monitoring response to behavioral pharmacotherapy (30, 43), or detecting affective burden in dogs with chronic medical conditions (32, 46), the field will need to move from group-level inferential designs toward indicators of individual diagnostic accuracy. Clinical translation will also require consideration of the professional context in which welfare measures are interpreted and applied. Veterinary behaviorists have been reported to place greater importance on psychological aspects of companion-animal welfare than veterinarians working in other areas (56), highlighting the value of standardized training and interpretation if affective-state measures are to be incorporated into clinical practice. Plausible benchmarks include adequate test–retest reliability over clinically meaningful intervals [substantially exceeding the values currently documented in Wilson et al. (28)], pre-specified standardized protocols covering apparatus geometry, training criterion, latency operationalization, and analytic pipeline, and reporting of sensitivity, specificity, and predictive values against an external reference such as validated clinical assessment, behavioral questionnaires, or treatment response. A standardized canine JBT protocol meeting these criteria, ideally co-developed across laboratories and validated in multi-site studies, would be a realistic medium-term target and would substantially expand the practical utility of the paradigm beyond its current research-only role.

### 4.7 Limitations of this review

Several limitations should be acknowledged. First, the heterogeneity of outcome measures prevented meta-analysis, limiting the precision of effect size estimates. While we qualitatively synthesized directionality and effect sizes where reported, quantitative synthesis would strengthen conclusions. Second, publication bias and small-study effects were not formally assessed. The included corpus is dominated by relatively small samples (median *N* = 30 across the 15 experimental studies), and several manipulations are represented by only one or two studies; this combination of small N and few studies per intervention is precisely the configuration in which selective publication of statistically significant findings can inflate apparent effect sizes. Formal quantitative assessment (e.g., funnel plots, Egger’s regression) was not feasible because too few studies report comparable effect-size estimates with their associated uncertainty. Even so, several qualitative indicators are consistent with possible small-study inflation: the very large oxytocin effect reported by Kis et al. (30) in approximately 16 dogs per cell, the contrast between strong single-study findings and null replications within the same intervention class (e.g., enrichment), and the concentration of significant results at small N. Future work should report effect sizes with confidence intervals, pre-register hypotheses, and ideally include null-result preprints, so that quantitative bias-assessment tools become applicable. Third, our assessment of methodological quality was limited to risk of bias in experimental studies; observational study quality assessment would have strengthened bias appraisal. Fourth, the primary search was limited to Scopus and PubMed, although a supplementary search of Web of Science Core Collection did not identify additional eligible studies within the original search period. Relevant studies indexed only in other databases, such as PsycINFO or CAB Abstracts, may nevertheless have been missed. The protocol for this review was not pre-registered, which we acknowledge as a limitation. Finally, the review’s scope was limited to published peer-reviewed literature in English; gray literature and non-English publications could contain relevant evidence.

## 5 Conclusion

The Judgment Bias Task has substantial promise as a measure of cognitive-affective state in dogs, demonstrated by consistent effects of some experimental manipulations and associations with relevant observational conditions. However, the current evidence base does not yet support JBT as a fully valid and reliable measure for either research or applied welfare assessment. Critical limitations include spatial memory confounds, poor test–retest reliability, methodological heterogeneity, limited attention to mechanism, and frequent methodological biases in experimental studies.

The finding that some deliberate experimental manipulations produce measurable JBT shifts addresses RQ2 affirmatively, though heterogeneous null results indicate that not all hypothesized affective manipulations reliably influence task performance. Observational and quasi-experimental associations with previous training methods provide complementary evidence but do not establish causality. The answer to RQ1 (whether the JBT yields valid and reliable evidence of affective state) is more qualified: JBT provides potentially valid evidence under certain methodological conditions, but substantial methodological barriers currently limit its reliability and validity for routine application. Substantial methodological standardization, explicit attention to confounding and mechanisms, and expanded convergent validity research are prerequisites for wider application.

Future research directions should prioritize standardization of spatial paradigms, systematic investigation of mechanism and confounds, test–retest reliability assessment, and multimodal convergent validity studies. The field would benefit from pre-registration, transparent reporting, and expanded investigation of non-spatial paradigms. With these methodological improvements, the JBT may yet become a valuable tool for understanding affective states in dogs and assessing the welfare impacts of housing, training, and environmental conditions. Until these improvements are implemented, however, cautious interpretation and complementary multi-method approaches remain advisable for practitioners and researchers alike.

## Data Availability

The original contributions presented in the study are included in the article/supplementary material, further inquiries can be directed to the corresponding author/s.
